# Endoscopic ultrasound-guided tissue acquisition using a novel Franseen needle for ampullary gangliocytic paraganglioma

**DOI:** 10.1055/a-2643-8870

**Published:** 2025-07-15

**Authors:** Yuichi Suzuki, Haruo Miwa, Kazuki Endo, Ritsuko Oishi, Hiromi Tsuchiya, Manabu Morimoto, Shin Maeda

**Affiliations:** 1 26437 Gastroenterological Center, Yokohama City University Medical Center, Yokohama, Japan; 2 Department of Gastroenterology, Yokohama City University Graduate School of Medicine, Yokohama, Japan

Endoscopic ultrasound-guided tissue acquisition (EUS-TA) is a widely used technique for diagnosing various gastrointestinal lesions. Regarding the needles for EUS-TA, the Acquire S (Boston Scientific, Marlborough, Massachusetts, USA) is a newly developed Franseen needle with a sharp and tapered stylet that can improve puncture performance while ensuring adequate tissue acquisition ( Fig. 1 ). EUS-TA has also been reported to be useful in the diagnosis of ampullary tumors 1 . Among these, gangliocytic paraganglioma (GP) is known as a rare submucosal tumor that predominantly occurs in the second part of the duodenum and periampullary region 2 . Histopathological diagnosis of GP requires evidence of three cellular components: epithelioid cells, spindle cells, and ganglion cells 3 . Preoperative diagnosis of GP by EUS-TA is limited due to insufficient specimen volume 4 . We report a case in which GP was preoperatively diagnosed by EUS-TA using the Acquire-S ( Video 1 ).

**Fig. 1:**
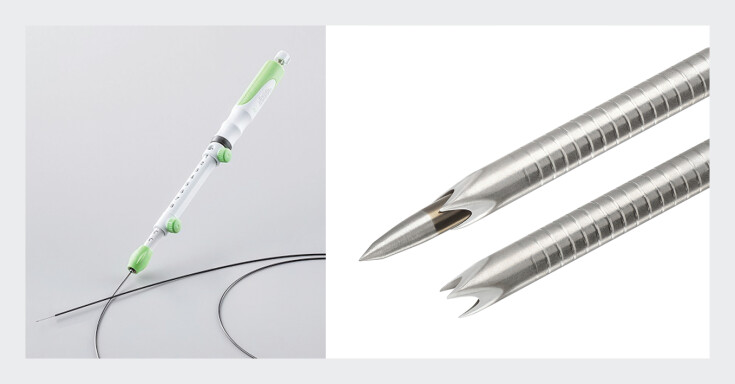
A novel Franseen needle (Acquire-S) has a sharp and tapered stylet for ease of puncture when performing endoscopic ultrasound-guided tissue acquisition (EUS-TA). The tapered stylet can be used in the advanced or retracted position based on the physician's preference. Source: Boston Scientific Corporation.

Ampullary gangliocytic paraganglioma was preoperatively diagnosed by endoscopic ultrasound-guided tissue acquisition using the Acquire S needle.Video 1

A 57-year-old woman was referred to our hospital due to epigastric pain and an ampullary tumor. Blood tests showed a mild elevation of hepatobiliary enzymes. Contrast-enhanced computed tomography suggested a hypervascular tumor located from the second to the third part of the duodenum. Endoscopic findings revealed a pedunculated, non-exposed ampullary tumor extending from the papilla to the third part of the duodenum ( Fig. 2 ). As the mucosal biopsy did not yield a definitive pathological diagnosis, EUS-TA was performed using a 22G Acquire S needle ( Fig. 3 ). Despite a soft and highly mobile tumor, the tumor was successfully punctured on the first pass. Histopathological examination confirmed the presence of the three cellular components, leading to the preoperative diagnosis of GP ( Fig. 4 ). Subsequently, a pancreaticoduodenectomy was performed, and the final diagnosis was also GP.

**Fig. 2:**
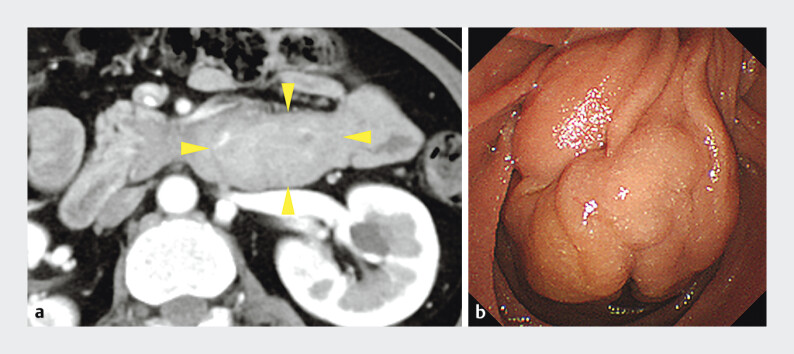
**a** Contrast-enhanced computed tomography suggested a hypervascular tumor (yellow arrowheads) located from the second to the third part of the duodenum. **b** Endoscopic findings revealed a pedunculated, non-exposed ampullary tumor measuring approximately 50 mm in diameter, extending from the papilla to the third part of the duodenum.

**Fig. 3:**
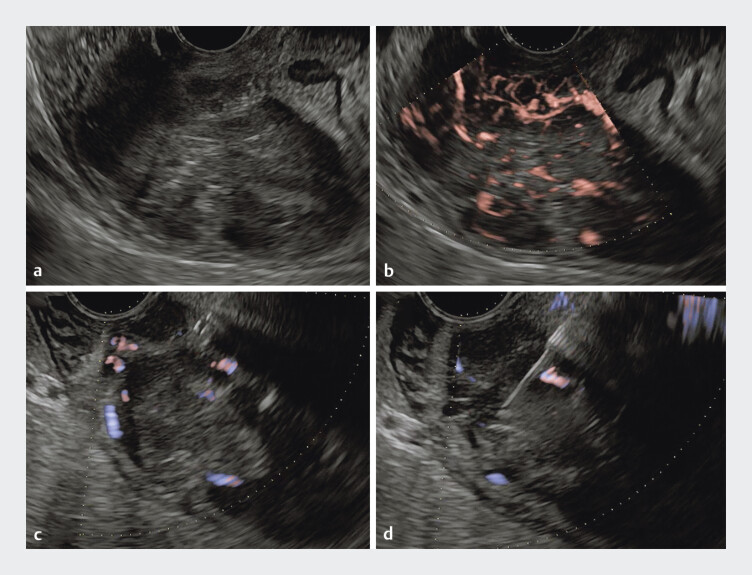
Ultrasonographic images of EUS-TA. **a** B-mode showed a large, hypoechoic tumor located from the second to the third part of the duodenum. **b** Detective flow imaging showed a hypervascular tumor. **c** Identification of the puncture route in areas with sparse vascularization on eFLOW. **d** EUS-TA was successfully performed using a 22G Acquire S needle with the slow-pull technique.

**Fig. 4:**
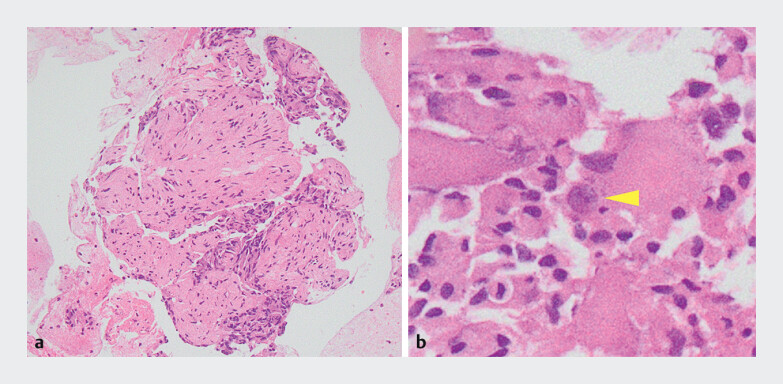
Histopathological evaluation of the EUS-TA specimens. **a** A large number of epithelioid cells and spindle cells can be detected (hematoxylin and eosin (HE) staining, ×100). **b** Ganglion cells can be detected under high magnification (yellow arrowhead, HE staining, ×400).

To the best of our knowledge, this is the first reported case of EUS-TA using the Acquire S. The Acquire S is useful for challenging cases in EUS-TA, such as highly mobile lesions.

Endoscopy_UCTN_Code_TTT_1AS_2AD

## Notes

Endoscopy E-Videos https://eref.thieme.de/e-videos
*E-Videos* is an open access online section of the journal *Endoscopy* , reporting on interesting cases and new techniques in gastroenterological endoscopy. All papers include a high-quality video and are published with a Creative Commons CC-BY license. Endoscopy E-Videos qualify for HINARI discounts and waivers and eligibility is automatically checked during the submission process. We grant 100% waivers to articles whose corresponding authors are based in Group A countries and 50% waivers to those who are based in Group B countries as classified by Research4Life (see: https://www.research4life.org/access/eligibility/ ). This section has its own submission website at https://mc.manuscriptcentral.com/e-videos .
